# Reactions of cisplatin and oxaliplatin with penicillin G: implications for drug inactivation and biological activity

**DOI:** 10.1007/s00775-022-01958-z

**Published:** 2022-09-25

**Authors:** Fang-Xin Wang, Ivan Prokes, Lijiang Song, Huayun Shi, Peter J. Sadler

**Affiliations:** 1 Department of Chemistry, University of Warwick, Coventry, CV4 7AL UK; 2 Department of Chemistry, Sun Yat-Sen University, Guangzhou, 510275 China

**Keywords:** Cisplatin, Oxaliplatin, Penicillin G, Degradation, Chemotherapy

## Abstract

Determination of the toxicity of compounds toward cancer cells is a frequent procedure in drug discovery. For metal complexes, which are often reactive prodrugs, care has to be taken to consider reactions with components of the cell culture medium that might change the speciation of the metal complex before it is taken up by the cells. Here, we consider possible reactions between the clinical platinum drugs cisplatin and oxaliplatin with penicillin G, an antibiotic added routinely to cell culture media to prevent bacterial contamination. Platinum has a high affinity for ligands with sulfur donors. Penicillin G is an unstable thioether that degrades in a range of pathways. Nuclear magnetic resonance (NMR) and UV–Vis absorption spectroscopic studies show that reactions with cisplatin can occur within minutes to hours at 310 K, but more slowly with oxaliplatin. The identities of the Pt- adducts were investigated by mass spectrometry. The marked effect on cytotoxicity of co-incubation of cisplatin with penicillin G was demonstrated for the HeLa human cervical cancer cell line. These studies highlight the possibility that reactions with penicillin G might influence the cytotoxic activity of metal complexes determined in culture media.

**Graphical abstract:**

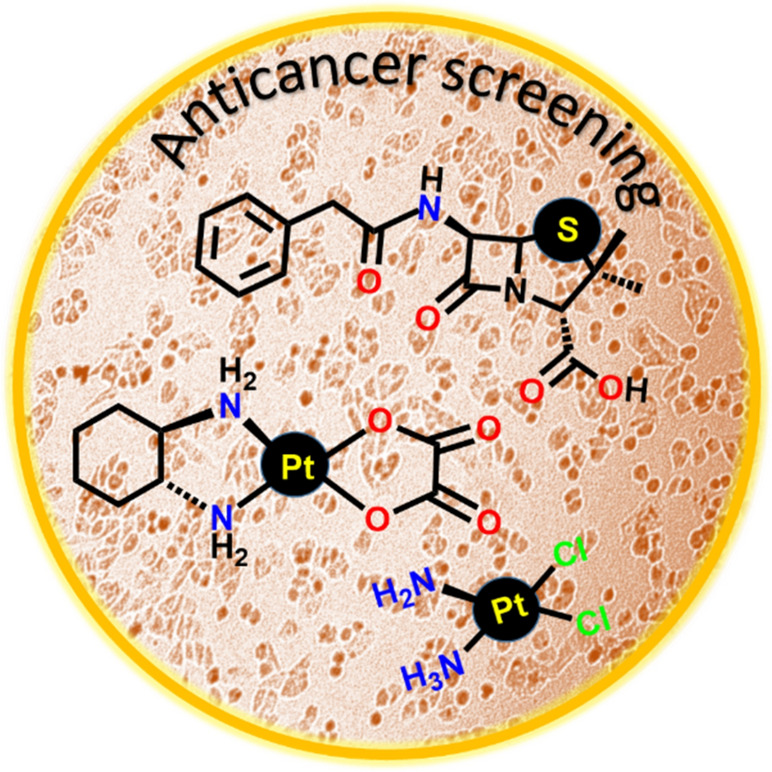

**Supplementary Information:**

The online version contains supplementary material available at 10.1007/s00775-022-01958-z.

## Introduction

Modern drug discovery often starts with the choice of a protein/enzyme target and design of compounds which bind strongly to that target. An ideal druggable target is only present in cancer cells, or is mutated, and not found in normal healthy cells [1]. This strategy can be successful, but relies on the subsequent ability of the compound to be taken up by cells and reach the target in vivo. An alternative approach is phenotypic screening, which does not require much prior knowledge of a target, and often begins with cytotoxicity assays using cells in culture [2]. This can lead to the discovery of novel targets, and also allows multitargeting, a potentially powerful strategy for combatting resistance.

In phenotypic anticancer screening, it is common to search for hits by determination of the cytotoxicity of compounds toward cancer cells in culture, either 2D cultures or 3D spheroid cultures [3]. This involves dissolving the compounds in a suitable solvent and adding them to cells in the culture medium, typically for a period of 24 h followed by removal of the compound, replacement with fresh medium, and allowing cell growth to continue for a further 72 h. Such protocols vary between laboratories, with some using continuous exposure of the cells to the compounds over periods as long as 96 h. Cell survival can then be determined by a variety of methods, usually colorimetric, e.g. using yellow tetrazolium salt (3-(4,5-dimethylthiazol-2-yl)-2,5-diphenyltetrazolium bromide (MTT) which is converted to purple formazan crystals by succinate dehydrogenase in the mitochondria of metabolically active cells, or sulforhodamine B (SRB), a dye which indicates the protein content in cells [4, 5].

The first step in screening is to dissolve the compound in a suitable solvent. Routinely dimethyl sulfoxide (DMSO) is a common solvent for compounds with poor aqueous solubility, but care has to be taken since DMSO is a good ligand for Pt(II) binding, especially the S atom of DMSO, although O-binding can also occur. To avoid decomposition, fresh solutions of cisplatin need to be used quickly, or preferably DMSO avoided altogether [6], since chloride displacement can be followed by NH_3_ release due to the high *trans* effect of sulfur [7]. Fortunately cisplatin, *cis*-[PtCl_2_(NH_3_)_2_], and especially oxaliplatin, [Pt(oxalate)(*1R,2R*-diaminocyclohexane)] {[Pt(dach)(oxalate)]}, react with DMSO only slowly. Chemically defined media, such as Roswell Park Memorial Institute 1640 medium (RPMI-1640), are often used for cells in suspension and Dulbecco’s minimum essential medium (DMEM) for adherent cells. Also necessary growth factors (e.g. insulin, vascular endothelial growth factor, and epidermal growth factor receptor) and other substances (e.g. heparin and non-essential amino acids) are added to cell culture media to maintain proliferation and phenotypes of specific cell lines [3]. It is common to supplement these defined media with fetal calf serum (typically 5–20%, v/v) and the antibiotics penicillin G and streptomycin (Pen/Strep).

**Scheme 1:**
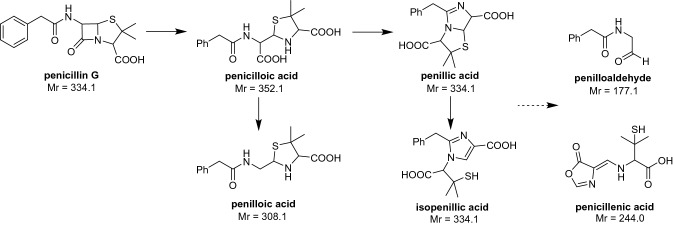
Decomposition pathways for penicillin G in water [14]. Mr = relative molecular mass

Pen/Strep are widely used as antibiotics in cell culture to prevent the contamination by Gram-positive and Gram-negative bacteria. Genomic screening has indicated that Pen/Strep can induce changes in gene expression. Several pathways including insulin response, fatty acid activation, mitochondrial l-carnitine shuttles, and unfolded protein response are affected by Pen/Strep in living cells [8]. Also they can affect the differentiation of stem cells and cancer cell lines, as well as cell cycle regulation [9]. Trace metabolites from penicillin degradation can cause hypersensitivity in patients with allergy problems, as the unstable β-lactam undergoes rapid self-catalytic hydrolysis followed by molecular rearrangement and degradation [10]. Consequently, it is suggested that commercial or self-prepared Pen/Strep should be stored away from light at 4 ℃ before use, or at − 20 ℃ for longer periods. However, this degradation in stock solutions or culture media appears to be less significant. Several metal ions (e.g. Zn^2+^ and Cd^2+^) have been reported to catalyze penicillin degradation [11]. The thioether group in penicillin G (Scheme 1) is a ‘soft’ nucleophile that might be expected to bind strongly to the ‘soft’ metal ion Pt(II), in an analogous manner to the side chain of the amino acid methionine [12]. Penicillin G is present in growth medium at 100 unit/mL (ca. 170 µM), a level which cannot be neglected with respect to Pt drug concentration [3]. To the best of our knowledge, interactions between platinum drugs and penicillin G have not been studied previously.

For clinical use, cisplatin (1 mg/ml sterile concentrate) is diluted with 0.9% saline solution (0.9 g NaCl per 100 mL H_2_O) or a mixture of 0.9% saline solution and 5% glucose solution (1:1) before administration, to prevent the release of labile chloride. Oxaliplatin for clinical infusion is usually diluted in 5% glucose solution, but is unstable and needs to be stored less than 48 h at 2–8 ℃ without light exposure if not administrated immediately [13]. In biological studies, cisplatin solutions (1–2.5 mM) are usually prepared freshly in phosphate buffered saline (PBS) or 0.9% saline solution. The final concentration of cisplatin in cell culture media is usually in the range 2–100 μM, with incubation times from several hours to days.

We estimate that over half of reported screening studies on metallodrugs use Pen/Strep (1%, v/v) in the culture medium, with a final concentration of penicillin G up to ca. 170 µM. We have borne these concentrations and conditions in mind in the present work for studies of reactions between cisplatin and penicillin G in solution. Using UV–Vis and nuclear magnetic resonance (NMR) spectroscopy, and mass spectrometry, we show that cisplatin readily forms adducts with hydrolyzed penicillin G in aqueous solution, and that the rate of reaction is influenced by the ionic strength of buffer solutions. In contrast, oxaliplatin is much less reactive under the same conditions. This study highlights the importance of considering possible reactions with common components of cell culture media in interpreting cell screening data.

## Materials and methods

### Materials

Na_2_HPO_4_·2H_2_O, NaH_2_PO_4_, NaCl and NaOH were purchased from Merck, USA. Cisplatin, oxaliplatin and penicillin G potassium were of USP grade and purchased from Merck. Fresh solutions of penicillin G potassium, cisplatin and oxaliplatin were prepared just before use. Na_2_HPO_4_ (10 mM) and NaH_2_PO_4_ (1.8 mM) were dissolved in the deionized water (15 MΩ/cm) to prepare the phosphate buffer (PB, pH 7.4 adjusted by NaOH). The concentration of chloride in PBS was 150 mM. The compositions and conditions for samples **a**–**i** studied in this work are summarized in Table 1. Deuterated water for NMR was purchased from Merck. HeLa cells were obtained from Experimental Animal Center of Sun Yat-Sen University, China. Dulbecco's Modified Eagle Medium (DMEM) and fetal bovine serum (FBS) were purchased from Gibco, USA. The MTT assay kit was purchased from Beyotime, China.

**Table 1:** The composition of samples used in this work

Sample	Composition
**a**	Penicillin G (1 mM) in deionized water
**b**	Penicillin G (1 mM) in PB
**c**	Penicillin G (1 mM) in PBS
**d**	Cisplatin (1 mM) and penicillin G (1 mM) in deionized water
**e**	Cisplatin (1 mM) and penicillin G (1 mM) in PB
**f**	Cisplatin (1 mM) and penicillin G (1 mM) in PBS
**g**	Oxaliplatin (1 mM) and penicillin G (1 mM) in deionized water
**h**	Oxaliplatin (1 mM) and penicillin G (1 mM) in PB
**i**	Oxaliplatin (1 mM) and penicillin G (1 mM) in PBS

### Methods

#### UV–Vis absorption spectroscopy

UV–Vis absorption spectra were recorded on a Cary 300 UV–Vis spectrophotometer fitted with a PTP1 Peltier temperature controller. The sample was freshly prepared and placed in a 1 cm quartz cuvette at 310 K. The absorbance was recorded from 250 to 500 nm every hour for 72 h.

#### NMR spectroscopy

^1^H NMR spectra were acquired in 5 mm NMR tubes at 298 K on a Bruker Avance III (^1^H = 600 MHz) or a Bruker Avance III HD (^1^H = 500 MHz). The solvent was 10% D_2_O/90% H_2_O. ^1^H NMR chemical shifts were referenced to the residual solvent peak (*δ* = 4.79 ppm). WATERGATE or presaturation water suppression techniques were used. ^13^C *J*-modulated spin-echo NMR spectra were acquired in 5 mm NMR tubes at 298 K on a Bruker Avance III HD (^13^C = 126 MHz) with 2048 scans. The [^15^N-^1^H] HSQC NMR spectrum was recorded on a Bruker Avance II (^1^H = 700 MHz, ^15^N = 70.95 MHz) at 298 K. The spectrum was recorded using 2D HSQC correlation via double INEPT transfer optimized for ^1^*J*(N,H) = 73 Hz (*τ* = 1/2* J*). 16 transients were acquired. All data processing was carried out using MestReNova software.

#### Electrospray ionization mass spectrometry (ESI–MS)

Low resolution MS data were acquired on an Agilent 6130B single quadrupole instrument. The samples (**a**, **d** and **g**) were incubated at 310 K for 24 h, and diluted 100-fold before detection. The *m/z* range from 50 to 2500 was recorded in both negative and the positive ion modes. High resolution MS data were collected on a Bruker Compact Q-TOF instrument in positive ion mode.

#### Cell culture and cytotoxicity assay

HeLa cells were incubated at 310 K in DMEM culture medium with 10% (v/v) FBS. No Pen/Strep was added to the medium during cell proliferation. The cytotoxicity of samples (**d**, **f**, **g** and **i**) toward HeLa cells was evaluated in 96-well plates by the MTT assay. The sample was prepared immediately or incubated at 310 K for 24 h to study degradation, and then added into each well to a final concentration of 20, 50 and 100 μΜ, and incubated with cells for 20 h. The culture medium was carefully replaced by fresh DMEM culture medium. MTT solution (20 μL, 5 mg/mL) was added into each well, and incubated for 4 h. The medium was discarded, and the plate was washed with PBS. Formazan in cells was dissolved in DMSO (150 μL per well) for 30 min. The absorbance at 595 nm was recorded on an Infinite M200pro microplate reader (Switzerland).

## Results and discussion

### Degradation of penicillin G

Penicillin G is known to undergo complicated degradation by different pathways in aqueous solution at neutral pH (Scheme 1) [14]. Sample **a** (potassium penicillin G in water) shows a major base peak at *m/z* 333.1([M–K^+^]^–^) in negative ion mode, with a minor fragmentation peak at *m/z* 192.1 (Fig. S1). As shown in Fig. S2 and Table S1, penicillin G transforms to penicilloic acid (*m/z* 307.1 and *m/z* 351.1) after incubation for 24 h at 310 K, which subsequently forms penilloic acid (*m/z* 307.1 and *m/z* 229.3) and penillic acid (*m/z* 333.1 and *m/z* 289.1). Besides, isopenillic acid (*m/z* 333.1 and *m/z* 255.2) can arise from penillic acid, and both of them can degrade to penilloaldehyde (*m/z* 176.1 and *m/z* 145.1).

**Fig. 1:**
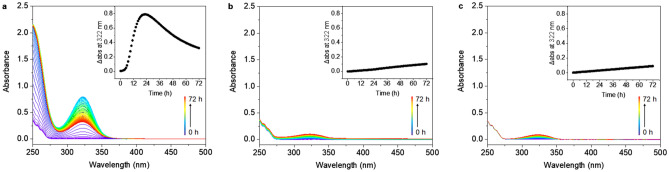
The UV–Vis absorption spectrum of penicillin G (1 mM) in **a** H_2_O (sample **a**), **b** PB (sample **b**), and **c** PBS (sample **c**) over a period of 72 h at 310 K. The insets show the change in absorbance (*Δ*abs) at 322 nm every hour

Penicillin G (**a**) quickly degraded to penicillenic acid, a chromophore that shows a peak absorption maximum at 322 nm in the UV–Vis spectrum [15], after 22 h (*τ*_1/2_ = 10.5 h) at 310 K (Fig. 1a), and then went through complicated further degradation [16]. Only ca. 13% of penicillin G (**b**) degraded to penicillenic acid at 310 K over 72 h (Fig. 1b), which is much slower than in water. The degradation of penicillin G (**c**) was further hindered by the presence of high concentrations of chloride, with ca. 11% penicillin G forming penicillenic acid in PBS over 72 h (Fig. 1c).

**Fig. 2:**
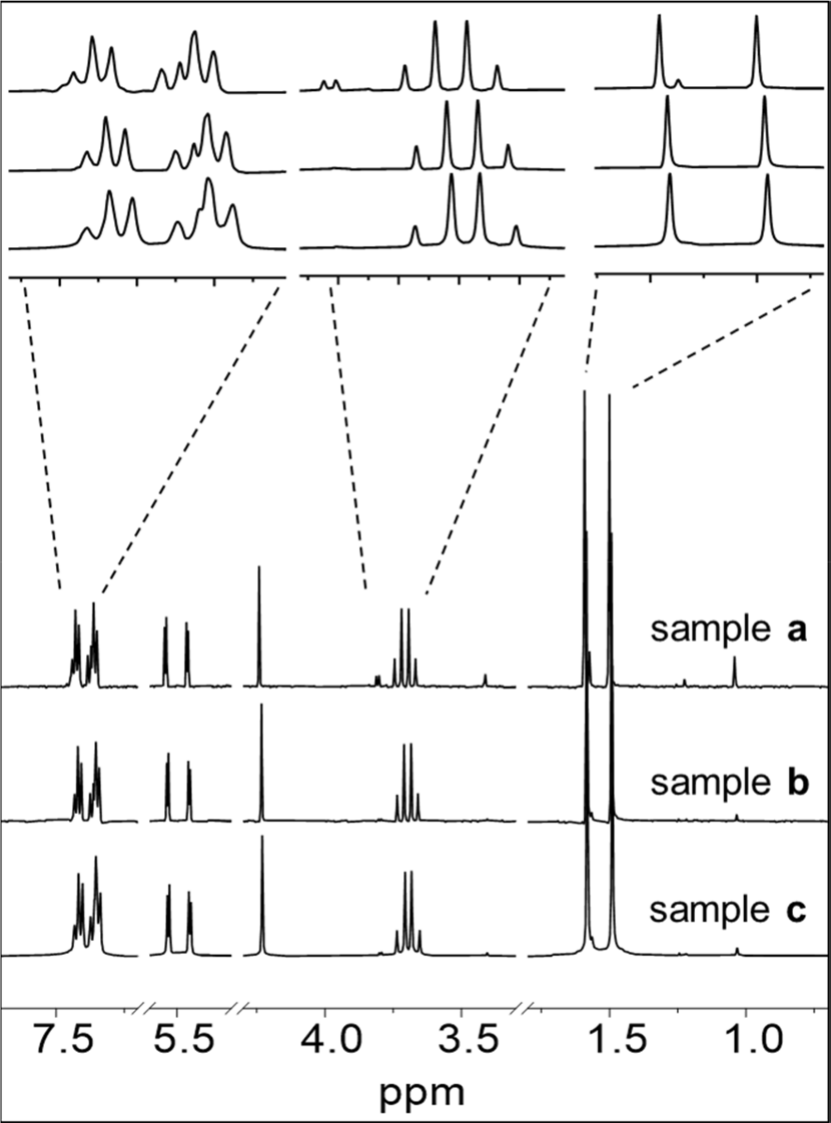
500 MHz ^1^H NMR spectra of samples **a** (in water), **b** (in PB), and **c** (in PBS) after incubation for 24 h at 310 K. The solvent was 90% H_2_O/10% D_2_O (v/v)

The degradation of penicillin G was also detected by NMR spectroscopy. Samples **a**-**c** in 90% H_2_O/10% D_2_O (v/v) were incubated for 24 h at 310 K. The ^1^H NMR spectrum (Fig. S3) and ^13^C *J*-modulated spin-echo NMR spectrum (Fig. S4) of penicillin G for signal assignment, are shown in the Supporting information. Penicillin G readily degrades in water, however, the amount of decomposition for sample **a** over this period was < 10% as judged by the minor set of peaks apparent from the overlapping methyl peaks for the five-membered thiazolidine ring at 1.4–1.6 ppm (Fig. 2). Samples **b** in PB and sample **c** in PBS degraded more slowly and gave rise to only minor additional peaks in ^1^H NMR spectra.

### Interaction of cisplatin and penicillin G

**Scheme 2:**
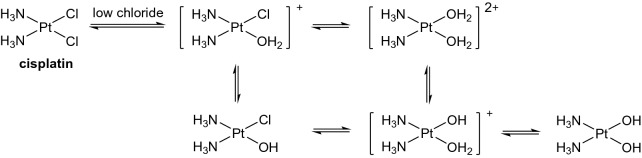
Aquation pathways for cisplatin and deprotonation of aqua adducts in aqueous solution [17]

The chloride ligands in cisplatin are labile, and mono-aqua and di-aqua species, which are more reactive than cisplatin itself, form in water (Scheme 2) [17]. Platinum(II) is a ‘soft’ metal ion which binds strongly to ‘soft’ ligands [18], and is therefore likely to bind to the soft sulfur atom in penicillin G and its degradants.

**Scheme 3:**
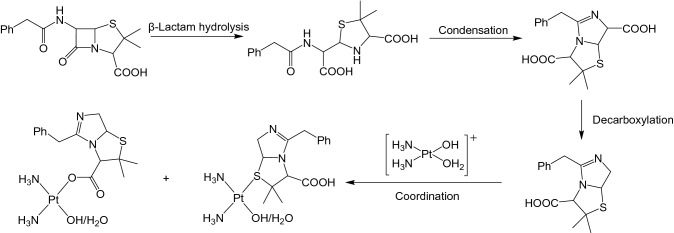
Possible coordination of {Pt(NH_3_)_2_}^2+^ to a penicillin G degradation product in water

**Fig. 3:**
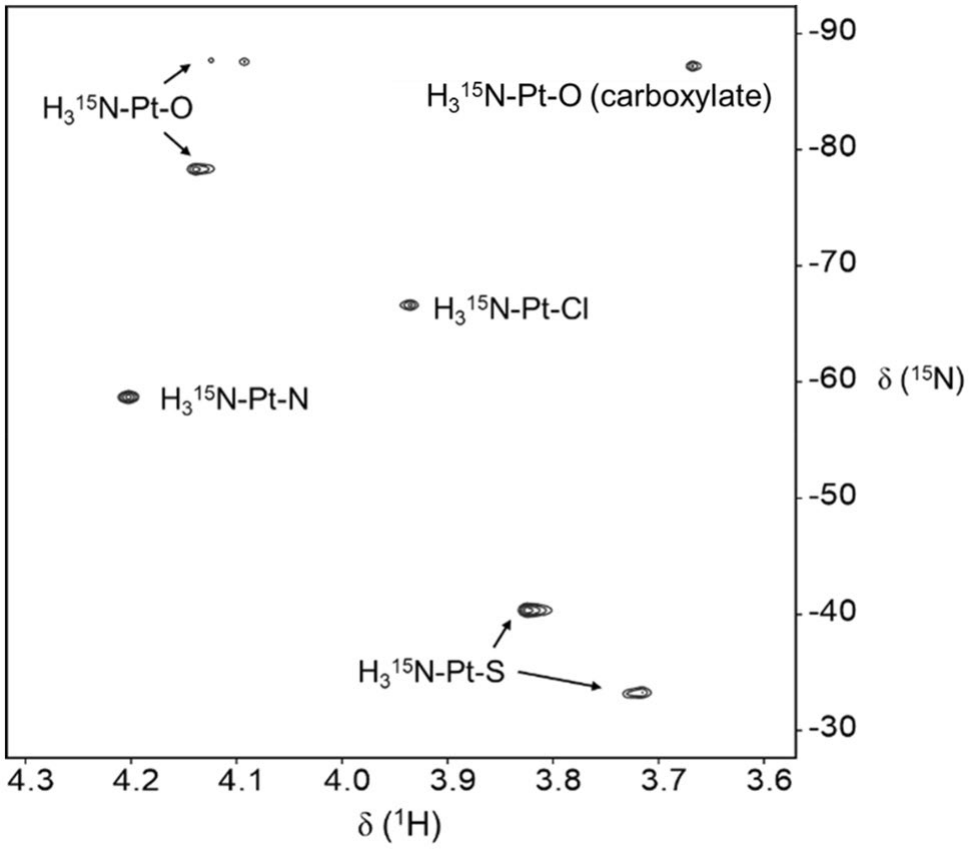
[^15^N-^1^H] 2D HSQC NMR spectrum of sample **d** after incubation at 310 K for 24 h. The ^15^ N NMR shifts are diagnostic of the type of ligand X in H_3_^15^N-Pt-X. Typically X can be O (*δ* − 75 to − 90 ppm), N/Cl (*δ* − 55 to − 70 ppm) or S (*δ* − 40 to − 50 ppm) [19]

First, we investigated the species in sample **d** (equimolar cisplatin and penicillin G) in water by low resolution MS. After incubation for 24 h at 310 K, a major cluster of peaks was detected in positive ion mode with the characteristic platinum isotope pattern shown over *m/z* 517–522 [(C_15_H_22_N_4_O_2_PtS)^+^] (Fig. S5), in which {Pt(NH_3_)_2_}^2+^ is probably coordinated to sulfur or carboxyl group of mono-decarboxylated penillic acid (Scheme 3). Evidence for S-coordination was obtained from [^15^N-^1^H] heteronuclear single quantum coherence (HSQC) 2D NMR studies of sample **d** (penicillin G and ^15^N-enriched cisplatin) (Fig. 3). The peaks at 3.83/− 40.4 ppm and 3.72/− 33.2 ppm have ^15^N chemical shifts in the expected range for ammonia *trans* to sulfur, H_3_^15^N–Pt–S [19]. A new peak appeared at 3.67/− 87.2 ppm, which may indicate the coordination of H_3_^15^N-Pt to O from the carboxylate group in penicillin G and its degradants [20].

**Fig. 4:**
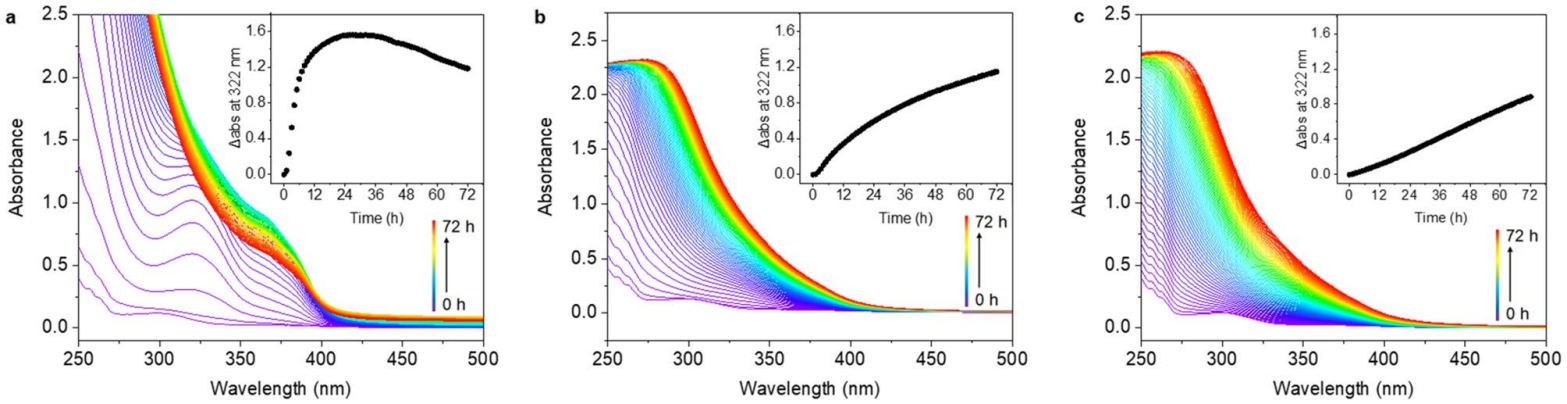
Changes in the UV–Vis absorption spectrum of cisplatin (1 mM) and penicillin G (1 mM) in** a** H_2_O (sample **d**),** b** PB (sample **e**), and** c** PBS (sample **f**), over a period of 72 h at 310 K. The insets are plots of the change in absorbance (*Δ*abs) at 322 nm versus time

The interaction between cisplatin and penicillin G was also studied by UV–Vis absorption spectroscopy. The absorbance of sample **d**, which contains equimolar cisplatin and penicillin G, increased rapidly in the range of 280–400 nm during the first 6 h and reached a maximum at 26 h at 310 K (Fig. 4a). As the half-life of penicillin G has shortened to 4 h, it seems likely that Pt species accelerate the degradation of penicillin G through coordination to the degradation products. Besides, the changes in the absorption spectrum of sample **d** differ from those of sample **a**. This also confirms that Pt influences the hydrolysis and decarboxylation of penicillin G, probably by coordination to carboxylate oxygen and sulfur sites. Further degradation of the Pt-based adduct was slower than that of penicillin G itself, suggesting that Pt stabilizes the adduct(s) from further degradation. Interestingly, samples **e** in PB and **f** in PBS show intense absorption from 250 to 450 nm (Fig. 4b, c), while cisplatin alone has only weak absorption in this region. This indicates that cisplatin interacts with penicillin G in phosphate buffer solutions, to the extent of Pt- adducts forming ca. 74% in PB and ca. 57% in PBS after 72 h.

**Fig. 5:**
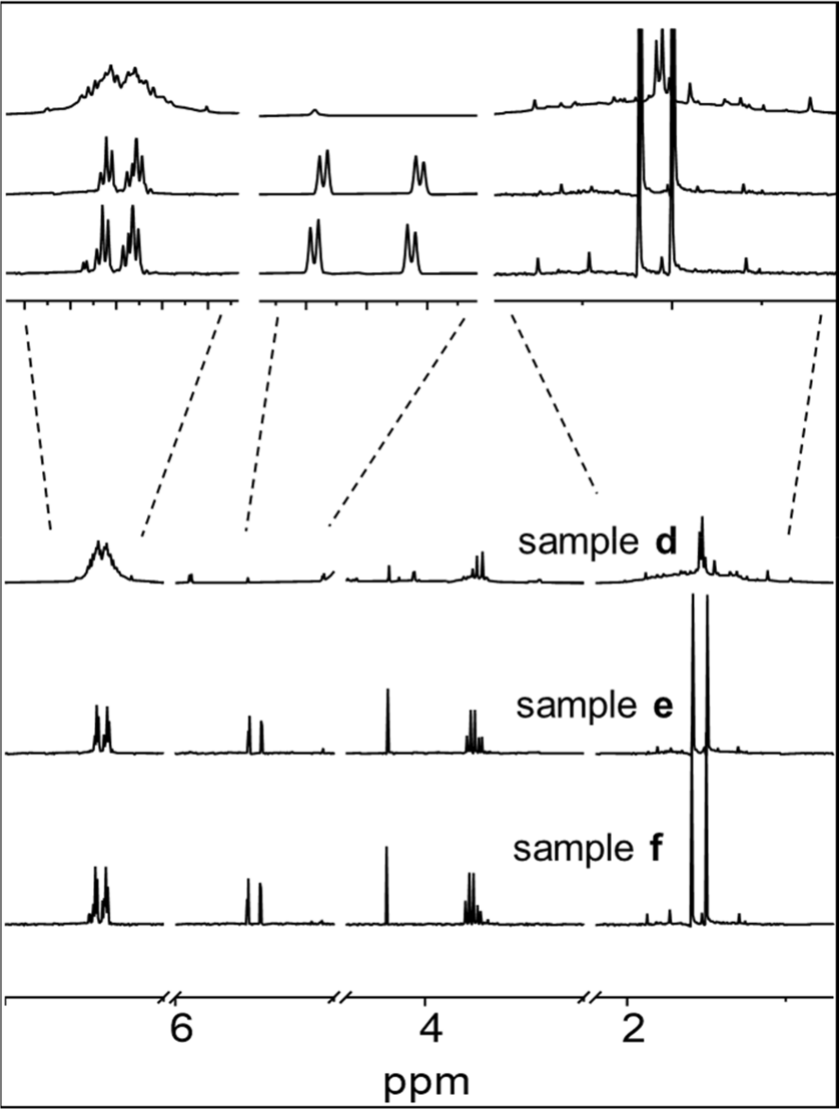
500 MHz ^1^H NMR spectra of cisplatin and penicillin G in water (sample** d**), in PB (sample** e**), and in PBS (sample** f**). Spectra were recorded after samples were incubated for 24 h at 310 K in 90% H_2_O/10% D_2_O (v/v)

The instability of sample **d** (cisplatin and penicillin G in water) after incubation at 310 K for 24 h is apparent in ^1^H NMR spectrum (**Fig. ****5**). The β-lactam peaks at *δ* 5.54 ppm (d, *J* = 3.9 Hz) and 5.46 ppm (d, *J* = 3.9 Hz), disappeared from sample **d** after 24 h, consistent with hydrolysis of the β-lactam. As β-lactam is a key component for antibacterial activity in the cell culture, its degradation in the presence of cisplatin or related Pt species is likely to deactivate penicillin G. The resonances at 7.30–7.45 ppm broaden to 7.1–7.6 ppm, indicative of the complexity of penicillin G degradants. The methyl resonances at 0.9–2.0 ppm clearly show the presence of broadened peaks, which also suggests the hydrolysis and further modifications to the thiazolidine ring. For samples **e** and **f**, minor proton resonances at 1.2–1.9 ppm were observed, suggesting that ca. 8% Pt- adducts are formed in the buffer solutions. However, assignment of these Pt- adducts is complicated. ^13^C signals of phenyl carbons (*δ* 127–130 ppm) are present in ^13^C NMR spectra, but other signals of penicillin G have disappeared (Fig. S6). This confirms that facile reactions of cisplatin and penicillin G can occur in aqueous solution.

### Interaction of oxaliplatin and penicillin G

**Scheme 4:**
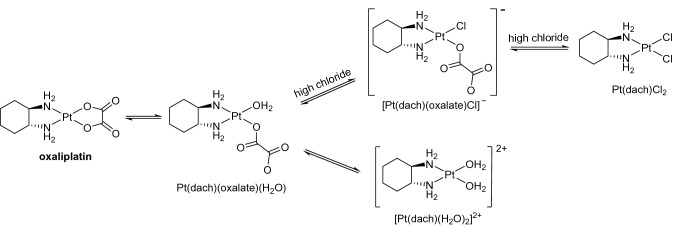
Hydrolysis pathways for oxaliplatin in aqueous solution

Oxaliplatin is more stable than cisplatin in aqueous media on account to the presence of chelated diaminocyclohexane (dach) and oxalate ligands. Some reactions can lead to very slow oxalate ring opening and formation of aqua complexes (Scheme 4), or the mono- and dichlorido species [Pt(dach)Cl_2_] in saline solution.

Sample **g** containing oxaliplatin (1 mM) and penicillin G (1 mM) was studied by low resolution mass spectrometry. After incubation for 24 h at 310 K, no peaks assignable to Pt- adducts were observed (Fig. S7), only peaks for oxaliplatin (*m/z* 357, 382, 420, 462) and its dimer (*m/z* 792). As expected, this indicates that oxaliplatin is more stable than cisplatin in water, and has little influence on the hydrolysis of penicillin G.

**Fig. 6:**
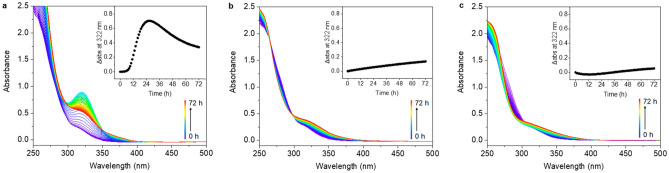
The UV–Vis absorption spectrum of oxaliplatin (1 mM) and penicillin G (1 mM) in **a** H_2_O (sample **g**), **b** PB (sample h), and **c** PBS (sample i) over a period of 72 h at 310 K. The insets show the increase in absorbance (*Δ*abs) at 322 nm versus incubation time

UV–Vis studies of sample **g**–**i** showed that reaction of oxaliplatin and penicillin G in deionized water is much slower than that of cisplatin, with the absorbance increase at 322 nm reaching a maximum at 27 h (*τ*_1/2_ = 15 h, Fig. 6a). The absorbances of samples** h** and **i** decrease at 265–293 nm, and increase over 293 nm, which probably indicates that oxaliplatin forms a monodentate oxalate platinum complex [Pt(dach)(oxalate)(H_2_O)] or aquated complex [Pt(dach)(H_2_O)_2_]^2+^ in PB (Fig. 6b). When oxalate in oxaliplatin is replaced by chloride, an apparent blue-shift from 300 to 267 nm occurs [21], and then interactions with penicillin G or its degradants can occur more readily (Fig. 6c).

**Fig. 7:**
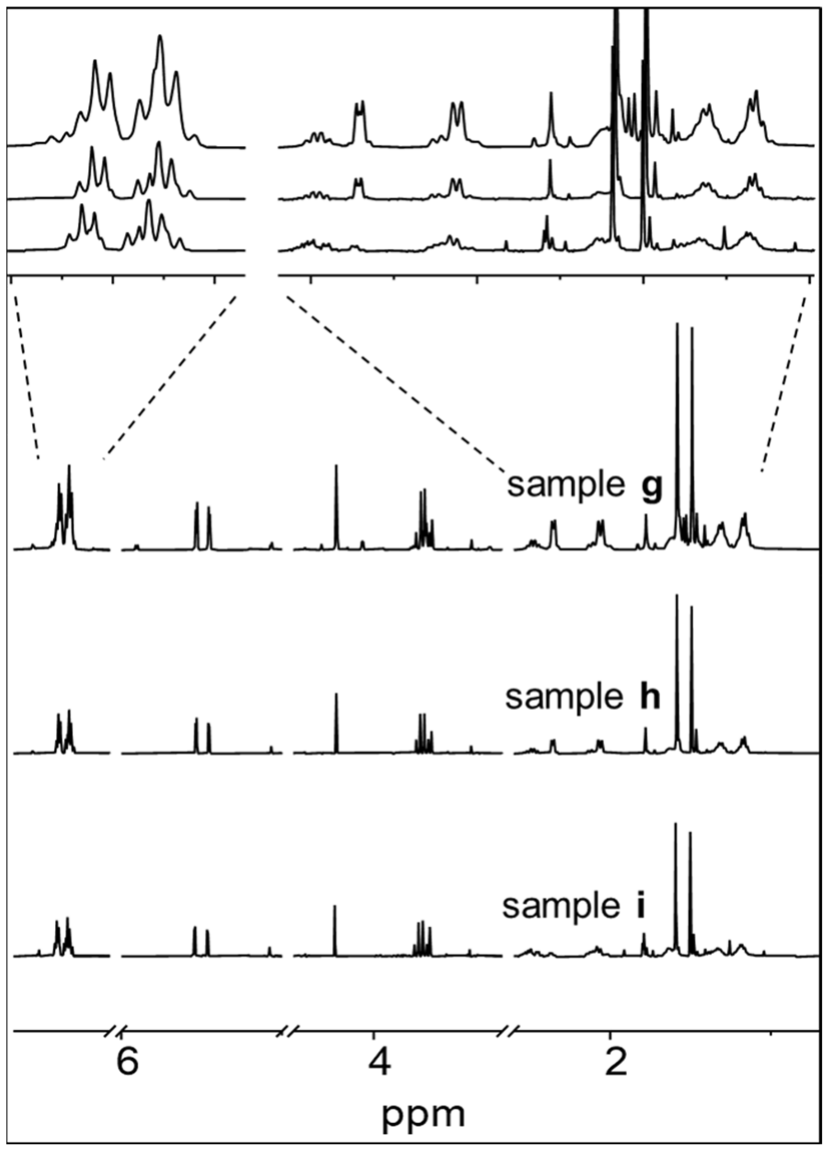
500 MHz ^1^H NMR spectra of oxaliplatin and penicillin G in water (sample** g**), in PB (sample** h**), and in PBS (sample** i**). Spectra were recorded after incubation of samples for 24 h at 310 K in 90% H_2_O/10% D_2_O (v/v)

^1^H NMR studies showed that oxaliplatin hydrolyzes faster in buffer solutions (samples **h** and **i**) than in water (sample **g**), as judged by the decreased intensities of the peaks of diaminocyclohexane on oxaliplatin from 1.1 ppm to 2.6 ppm (Fig. 7). The intensity of methyl resonances in penicillin G at 1.50 ppm and 1.59 ppm also decreased for samples **h** and **i**, indicating the faster degradation of penicillin G in the presence of oxaliplatin in buffer solutions. Oxaliplatin is unstable in high chloride-containing solutions, and its aqua species accelerate the degradation of penicillin G to some extent. The extent of degradation of penicillin G under the various conditions and coordination to Pt drugs are summarized in Table S2.

### Cytotoxicity and biological activity

**Fig. 8:**
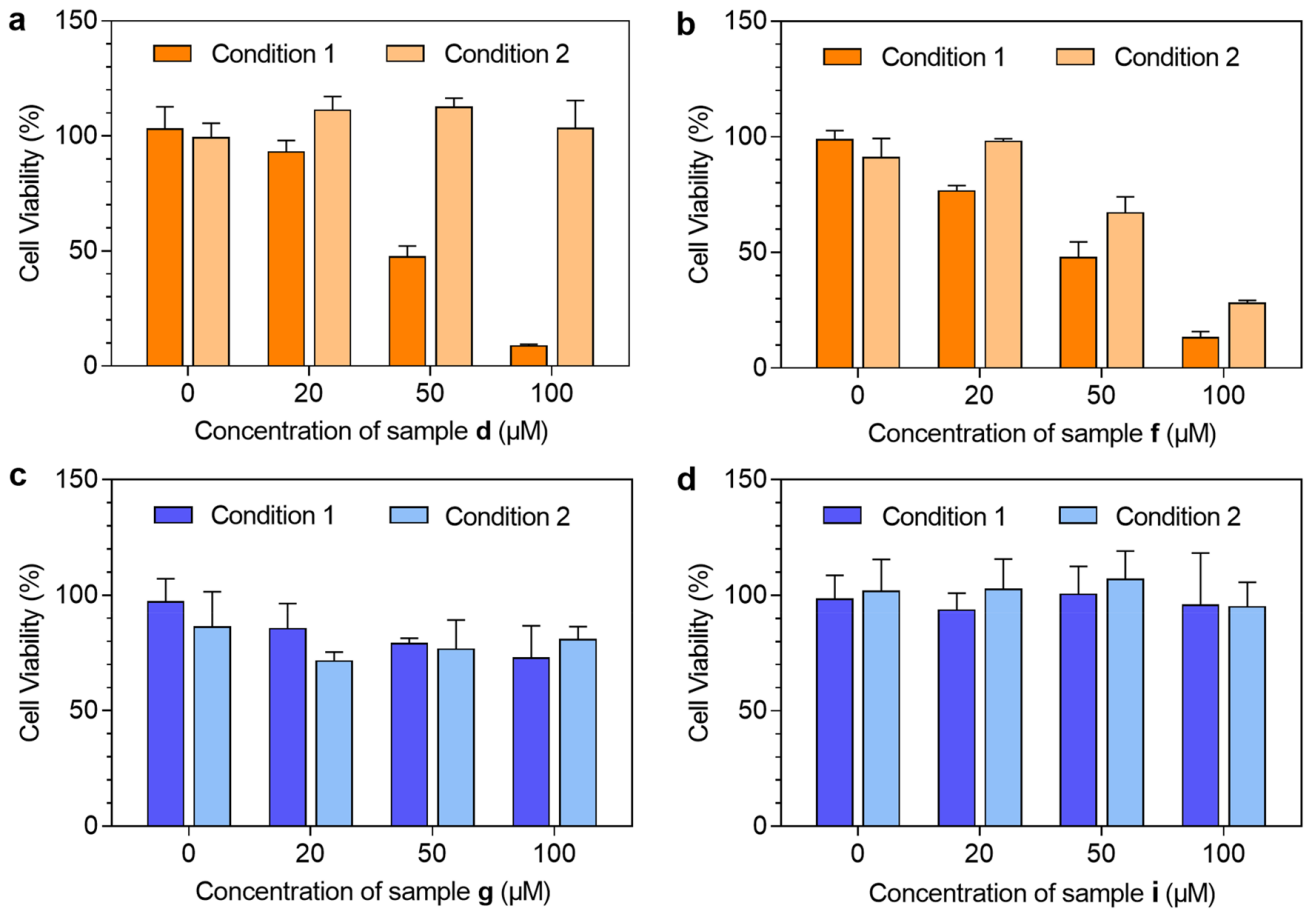
The cytotoxicity of (**a**) sample **d** (cisplain and penicillin G in water), (**b**) sample **f** (cisplatin and penicillin G in PBS), (**c**) sample** g** (oxaliplatin and penicillin G in water), and (**d**) sample** i** (oxaliplatin and penicillin G in PBS) toward HeLa cells for 24 h. Condition 1: sample was pre-incubated at 4 ℃ for 24 h before drug treatment. Condition 2: sample was pre-incubated at 310 K for 24 h before drug treatment

Cisplatin and oxaliplatin function as prodrugs in anticancer chemotherapy, so potential reactions with penicillin G in culture media could influence their in vitro anticancer cytotoxicity. Here, we chose commonly used HeLa cells derived from human cervical cancer cell line, to study the cytotoxicity. Sample **d** (cisplatin and penicillin G in deionized water), **f** (cisplatin and penicillin G in PBS), **g** (oxaliplatin and penicillin G in deionized water) and **i** (oxaliplatin and penicillin G in PBS) were freshly prepared and pre-incubated at 277 K or 310 K for 24 h. HeLa cells were treated with diluted samples (20–100 μM cisplatin or oxaliplatin with equimolar penicillin G) for 20 h, and cell viability was evaluated using the MTT cytotoxicity assay. Platinum drugs and penicillin G are stable at low temperature and undergo complicated hydrolysis and other interactions at normal body temperature. As suggested by the cytotoxicity data in Fig. 8a, sample **d** degrades extensively after incubation at 310 K for 24 h, indicative of cisplatin inactivation in the presence of penicillin G. The mixture of cisplatin and penicillin G in PBS (sample **f**) at 310 K also lost its cytotoxicity, attributable to the reaction of penicillin G with cisplatin even with the presence of high chloride concentrations (Condition 2, Fig. 8b). As expected, the cytotoxicity of sample **f** was less attenuated when the mixture was incubated at low temperature (277 K, Condition 1, Fig. 8b).

Oxaliplatin is less toxic than cisplatin, with an IC_50_ value of > 50 μM for this cell line. Unlike cisplatin, that induces cell death by interfering in DNA synthesis and arrests the cell cycle within S and G2/M phases at high doses, oxaliplatin mainly induces G1 phase cell cycle and ribosome biogenesis stress [22]. The mixture of oxaliplatin and penicillin G in water (sample **g**) shows similar cytotoxicity when pre-incubated at 277 K or 310 K for 24 h (Fig. 8c). When pre-incubated in PBS, the effect of drug degradation on cell death was not as evident as for cisplatin and penicillin G (Fig. 8d).

Since both cisplatin and oxaliplatin can react with penicillin G and its degradation products, the cytotoxicity of these drugs in biological tests might not be solely that induced by the intact metallodrugs, but also influenced by the Pt-adducts. This possibility needs to be considered in future studies of the activation of these prodrugs, as well as their anticancer activity and mechanism of action.

## Conclusions

Penicillin G, an antibiotic commonly added to cell culture media used for cytotoxicity screening, degrades by several pathways forming complicated mixtures of species. Here we show that cisplatin and oxaliplatin can interact with penicillin G and form a series of Pt-adducts. The cytotoxicity of Pt drugs toward cancerous HeLa cells decreased when pre-incubated with penicillin G in water or buffer solution. Many metallodrugs function as prodrugs, which undergo activation via different mechanisms (e.g. ligand substitution, redox reactions). Besides the stability of metallodrugs, attention should be paid to possible reactions with components of the cell culture medium or body fluids, as well as to drug co-administration, which may inactivate or even activate metallodrugs, and cause unexpected side effects in clinic chemotherapy. The possibility of such reactions occurring will need to be assessed in each laboratory for the particular screening conditions used, including consideration of cell-type, cell number, growth medium, incubation time, and the competitive rate of drug uptake into cells by passive or active (transporter-mediated) mechanisms.

## Supplementary Information

Below is the link to the electronic supplementary material.

Supplementary file1 (PDF 532 KB)

## Abbreviations

DMSO: Dimethylsulfoxide
DMEM: Dulbecco’s minimum essential medium
ESI–MS: Electrospray ionization mass spectrometry
HSQC: Heteronuclear single quantum coherence
MTT: (3-(4,5-Dimethylthiazol-2-yl)-2,5-diphenyltetrazolium bromide
NMR: Nuclear magnetic resonance
PBS: Phosphate buffered saline
PB: Phosphate buffer
RPMI-1640: Roswell Park Memorial Institute 1640 medium
SRB: Sulforhodamine B

## References

[1] Knowles J, Gromo G (2003). Target selection in drug discovery. Nat Rev Drug Discov.

[2] Moffat JG, Rudolph J, Bailey D (2014). Phenotypic screening in cancer drug discovery — past, present and future. Nat Rev Drug Discov.

[3] Anthony EJ, Bolitho EM, Bridgewater HE, Carter OWL, Donnelly JM, Imberti C, Lant EC, Lermyte F, Needham RJ, Palau M, Sadler PJ, Shi H, Wang FX, Zhang WY, Zhang Z (2020). Metallodrugs are unique: opportunities and challenges of discovery and development. Chem Sci.

[4] Stockert JC, Horobin RW, Colombo LL, Blázquez-Castro A (2018). Tetrazolium salts and formazan products in cell biology: viability assessment, fluorescence imaging, and labeling perspectives. Acta Histochem.

[5] Vichai V, Kirtikara K (2006). Sulforhodamine B colorimetric assay for cytotoxicity screening. Nat Protoc.

[6] Hall MD, Telma KA, Chang KE, Lee TD, Madigan JP, Lloyd JR, Goldlust IS, Hoeschele JD, Gottesman MM (2014). Say no to DMSO: dimethylsulfoxide inactivates cisplatin, carboplatin, and other platinum complexes. Cancer Res.

[7] Kerrison SJS, Sadler PJ (1977). Solvolysis of cis-[Pt(NH_3_)_2_Cl_2_] in dimethyl sulphoxide and reactions of glycine with [PtCl_3_(Me_2_SO)]^-^ as probed by ^195^Pt nuclear magnetic resonance shifts and ^195^Pt-^15^N coupling constants. Chem Commun.

[8] Ryu AH, Eckalbar WL, Kreimer A, Yosef N, Ahituv N (2017). Use antibiotics in cell culture with caution: genome-wide identification of antibiotic-induced changes in gene expression and regulation. Sci Rep.

[9] Skubis A, Gola J, Sikora B, Hybiak J, Paul-Samojedny M, Mazurek U, Łos MJ (2017). Impact of antibiotics on the proliferation and differentiation of human adipose-derived mesenchymal stem cells. Int J Mol Sci.

[10] Har D, Solensky R (2017). Penicillin and beta-lactam hypersensitivity. Immunol Allergy Clin N Am.

[11] Navarro PG, Blázquez IH, Osso BQ, Martínez de las Parras PJ, Puentedura MAIMN, García AAM (2003). Penicillin degradation catalysed by Zn(II) ions in methanol. Int J Biol Macromol.

[12] Norman RE, Ranford JD, Sadler PJ (1992). Studies of platinum(II) methionine complexes: metabolites of cisplatin. Inorg Chem.

[13] Lévi F, Metzger G, Massari C, Milano G (2000). Oxaliplatin. Clin Pharmacokinet.

[14] Aldeek F, Canzani D, Standland M, Crosswhite MR, Hammack W, Gerard G, Cook JM (2016). Identification of penicillin g metabolites under various environmental conditions using UHPLC-MS/MS. J Agric Food Chem.

[15] Schwartz MA, Tutt DE (1971). Model catalysts which stimulate penicillinase. V. The cycloheptaamylose-catalyzed hydrolysis of penicillins. J Am Chem Soc.

[16] Garlock EA, Grove DC (1950). The quantitative determination of benzylpenicillin by ultraviolet absorption. J Pharm Sci.

[17] Berners-Price SJ, Frenkiel TA, Frey U, Ranford JD, Sadler PJ (1992) Hydrolysis products of cisplatin: pK_a_ determinations via [^1^H, ^15^N] NMR spectroscopy. JCS Chem Comm, pp 789–791

[18] Wang X, Guo Z (2007). The role of sulfur in platinum anticancer chemotherapy. Anti-Cancer Agents Med Chem.

[19] Barnham KJ, Djuran MI, Murdoch PdS, Ranford JD, Sadler PJ (1996). Ring-opened adducts of the anticancer drug carboplatin with sulfur amino acids. Inorg Chem.

[20] Chen Y, Guo Z, Sadler PJ, Lippert B (1999). ^195^Pt and ^15^N NMR spectroscopic studies of cisplatin reactions with biomolecules. Cisplatin—chemistry and biochemistry of a leading anticancer drug.

[21] Zou Y, Biao L, Xu F, Liu R, Liu Z, Fu Y (2016). Structural study on the interactions of oxaliplatin and linear DNA. Scanning.

[22] Bruno PM, Liu Y, Park GY, Murai J, Koch CE, Eisen TJ, Pritchard JR, Pommier Y, Lippard SJ, Hemann MT (2017). A subset of platinum-containing chemotherapeutic agents kills cells by inducing ribosome biogenesis stress. Nat Med.

