# Supplementary material for: Reactions of cisplatin and oxaliplatin with penicillin G: implications for drug inactivation and biological activity
Source: J Biol Inorg Chem. 2022 Sep 25;27(8):695–704. doi: 10.1007/s00775-022-01958-z (PMC9653338; doi:10.1007/s00775-022-01958-z)
Supplement: Supplementary file 1 — Supplementary file1 (PDF 532 KB) [file 775_2022_1958_MOESM1_ESM.pdf]

Electronic Supplementary Material

Reactions of cisplatin and oxaliplatin with penicillin G: implications for drug inactivation and biological activity

Fang-Xin Wang,[1,2] Ivan Prokes,[1] Lijiang Song,[1] Huayun Shi,[1] Peter J. Sadler[1*]

1. Department of Chemistry, University of Warwick, Coventry, CV4 7AL, UK
2. Department of Chemistry, Sun Yat-Sen University, Guangzhou, 510275, China

Contents

Table S1. Penicillin G and its degradants in sample **a** detected by LTQ-MS

Table S2. Penicillin G degradation and Pt coordination in various aqueous solutions

Figure S1. ESI-MS spectrum of penicillin G

Figure S2. ESI-MS spectrum of penicillin G after degradation in water

Figure S3. ^{1}H NMR spectrum of penicillin G in 90% H_2O/10% D_2O (v/v).

Figure S4. ^{13}C J-modulated spin-echo NMR spectrum of penicillin G in 90% H_2O/10% D_2O (v/v)

Figure S5. High resolution ESI-MS spectrum of sample **d** after incubation at 310 K for 24 h

Figure S6. ^{13}C J-modulated spin-echo NMR spectrum of sample **a**, **d** and **g** in 90% H_2O/10% D_2O (v/v)

Figure S7. ESI-MS spectrum of oxaliplatin and penicillin G after incubation in deionized water at 310 K for 24 h

Table S1. Penicillin G and its degradants in sample **a** detected by LTQ-MS

Species	*m/z* and relative abundance [a]	
	Condition I [b]	Condition II [c]
penicillin G	333.1 (100)<br>192.1 (7)	333.1 (100)<br>192.1 (31)
penicillonic acid		351.1 (7)<br>307.1 (27)
penilloic acid		307.1 (27)<br>229.2 (3)
penillic acid		333.1 (100)<br>289.1 (63)
isopenillic acid		333.1 (100)<br>255.2 (7)
penicilloaldehyde		176.1 (30)<br>145.1 (10),

[a] The mass/charge ratios (*m/z*) are for deprotonated species with one negative charge observed in negative ion mode, and their abundances relative to penicillin G (set as 100) are shown in brackets.

[b] In Condition I, potassium penicillin G was dissolved in deionized water, and detected by LTQ-MS immediately.

[c] In Condition II, potassium penicillin G was dissolved in deionized water and incubated at 310 K for 24 h, and then detected by LTQ-MS.

Table S2. Penicillin G degradation and Pt coordination in various aqueous solutions

Aqueous solution	Penicillin G	Penicillin G and cisplatin	Penicillin G and oxaliplatin
Deionized water	100% degradants formed ($\tau_{1/2}$=10.5 h) [a]	100% Pt- adducts formed ($\tau_{1/2}$=4 h) [a]	18% Pt adducts formed [b]
PB	13% degradants formed [a]	74% Pt adducts formed [a]	43% Pt adducts formed [b]
PBS	11% degradants formed [a]	57% Pt adducts formed [a]	82% Pt adducts formed [b]

[a] Sample incubated at 310 K for 72 h and monitored by UV-vis spectroscopy.
[b] Sample incubated at 310 K for 24 h and monitored by ^{1}H NMR spectroscopy.

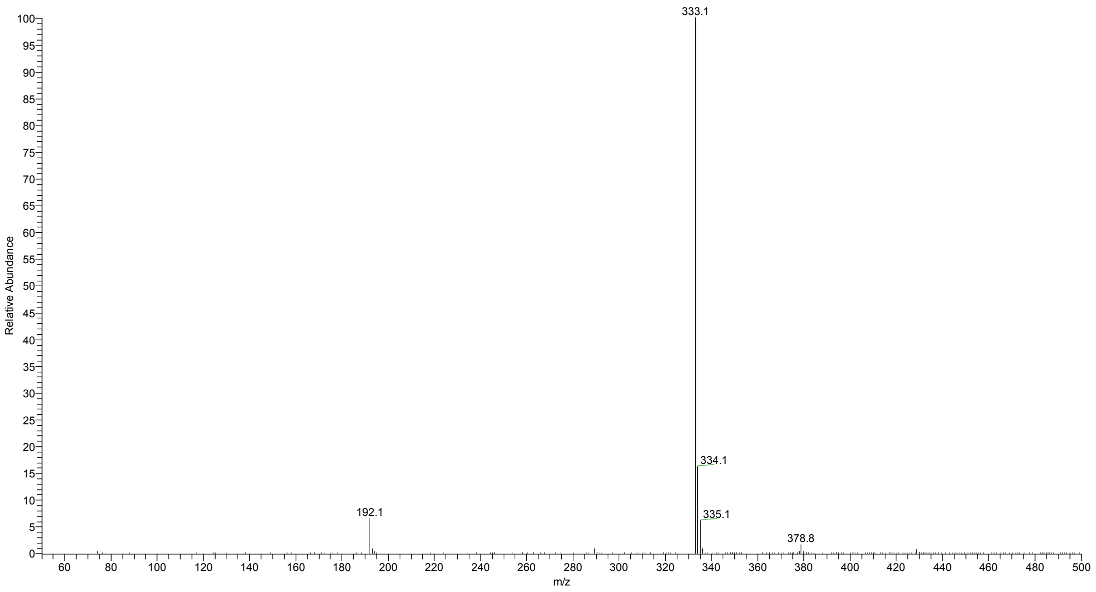

Figure S1. ESI-MS spectrum of potassium penicillin G in negative ion mode. The major ion (m/*z* 333.1) is $[M\text{-}K^{+}]^{-}$. Penicillin G potassium was dissolved in deionized water and then the spectrum recorded immediately by LTQ ESI-MS in negative-ion mode.

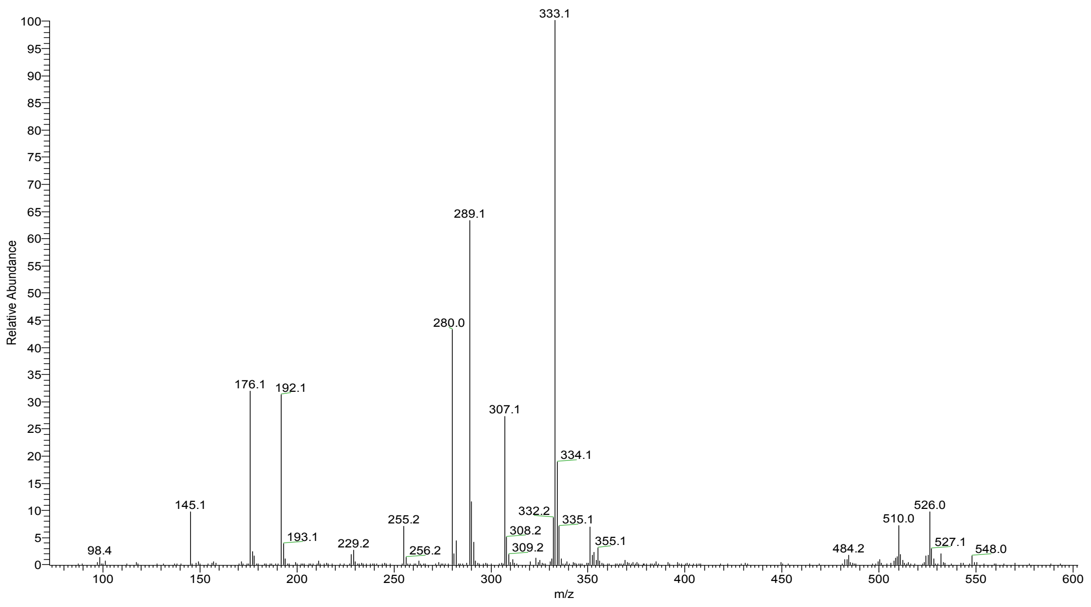

Figure S2. ESI-MS spectrum showing degradation of penicillin G in water. Penicillin G was dissolved in deionized water to 1 mM, and then incubated at 310 K for 24 h. Sample was diluted by 100-fold before LTQ ESI-MS in negative-ion mode was recorded.

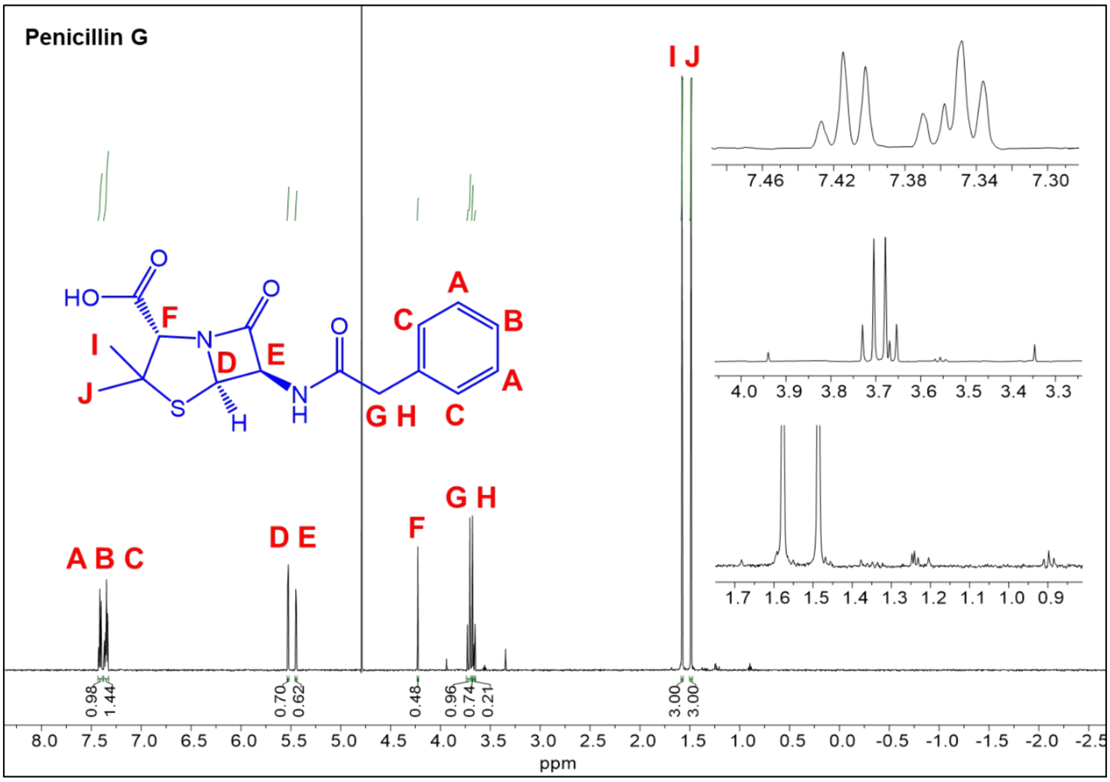

Figure S3. The ^{1}H NMR spectrum of penicillin G in 90% H_2O/10% D_2O (v/v).

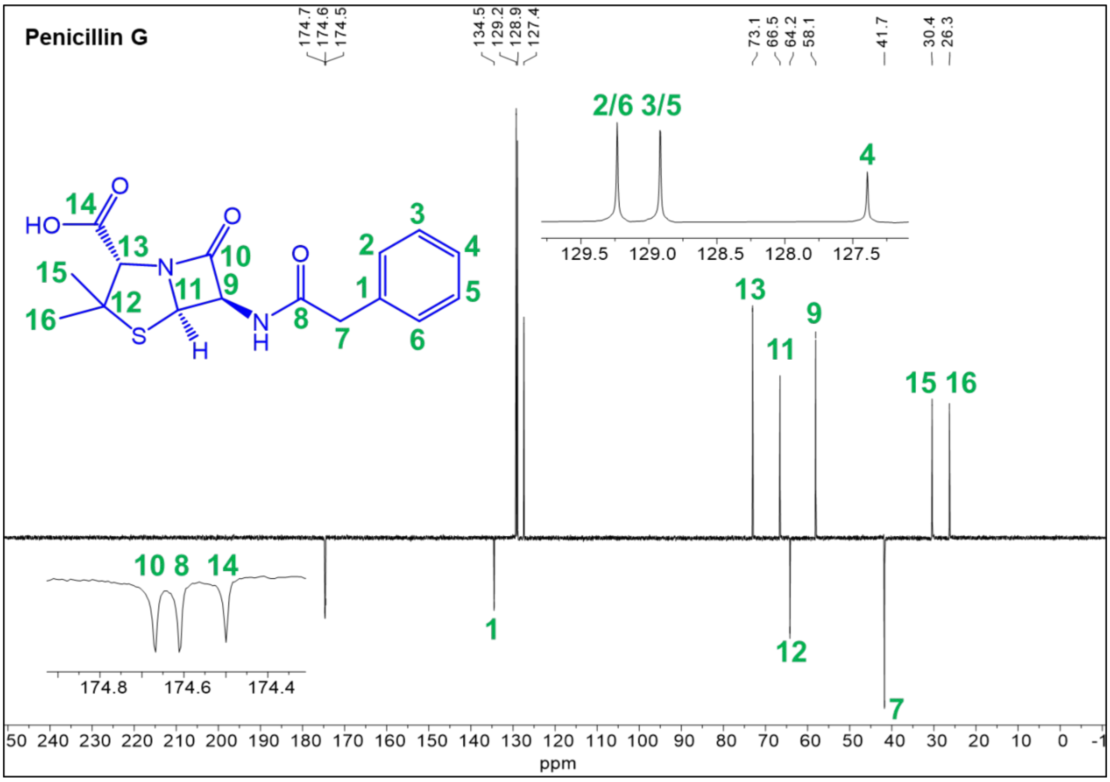

Figure S4. The ^{13}C J-modulated spin-echo NMR spectrum of penicillin G in 90% H_2O/10% D_2O (v/v).

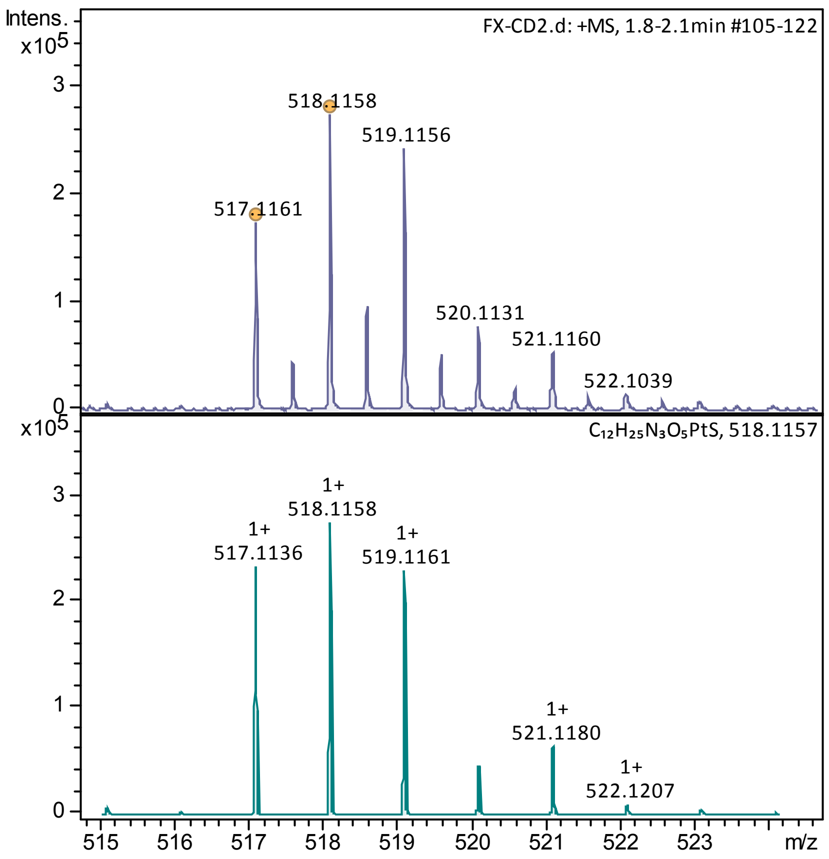

Figure S5. The mass spectrum of sample **d** after incubation at 310 K for 24 h. Top: observed mass spectrum. Bottom: simulated mass spectrum.

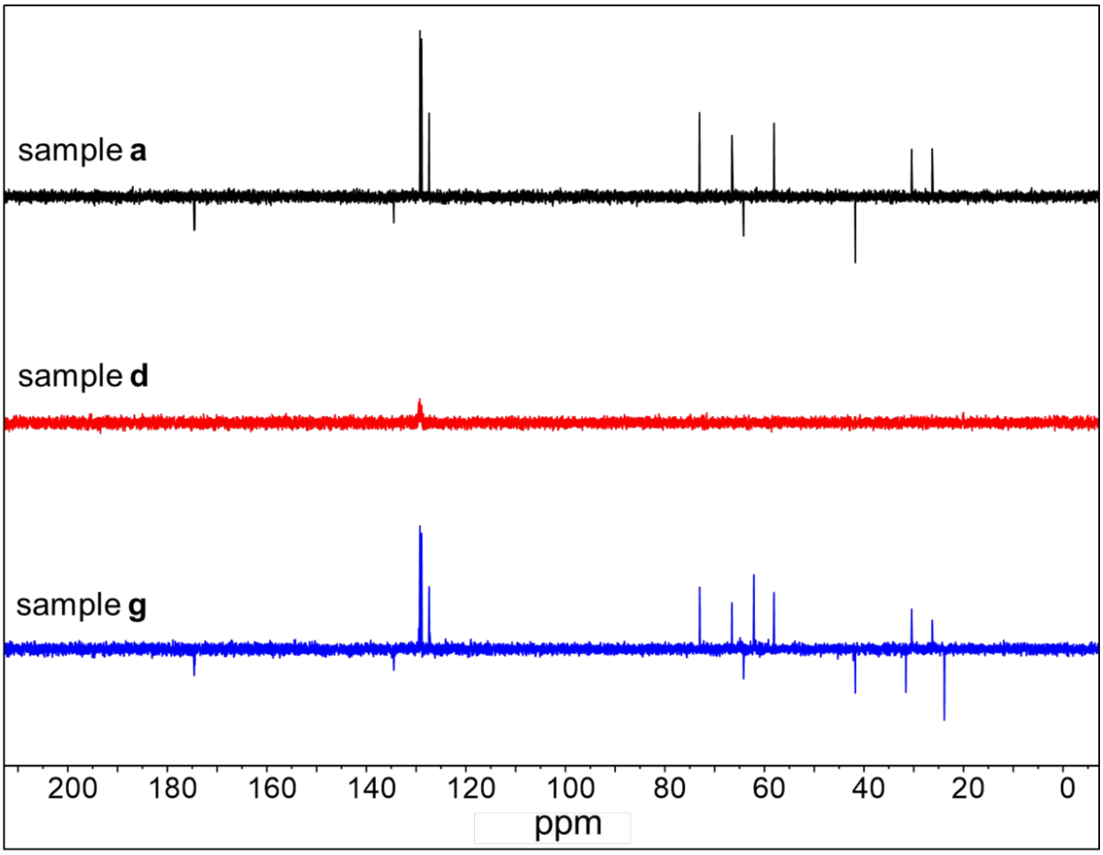

Figure S6. The ^{13}C J-modulated spin-echo spectra of sample **a** (1 mM penicillin G), sample **d** (1 mM cisplatin and 1 mM penicillin G), and sample **g** (1 mM oxaliplatin and 1 mM penicillin G) in 90% H_2O/10% D_2O (v/v). Samples were incubated at 310 K for 24 h before ^{13}C NMR spectra were recorded.

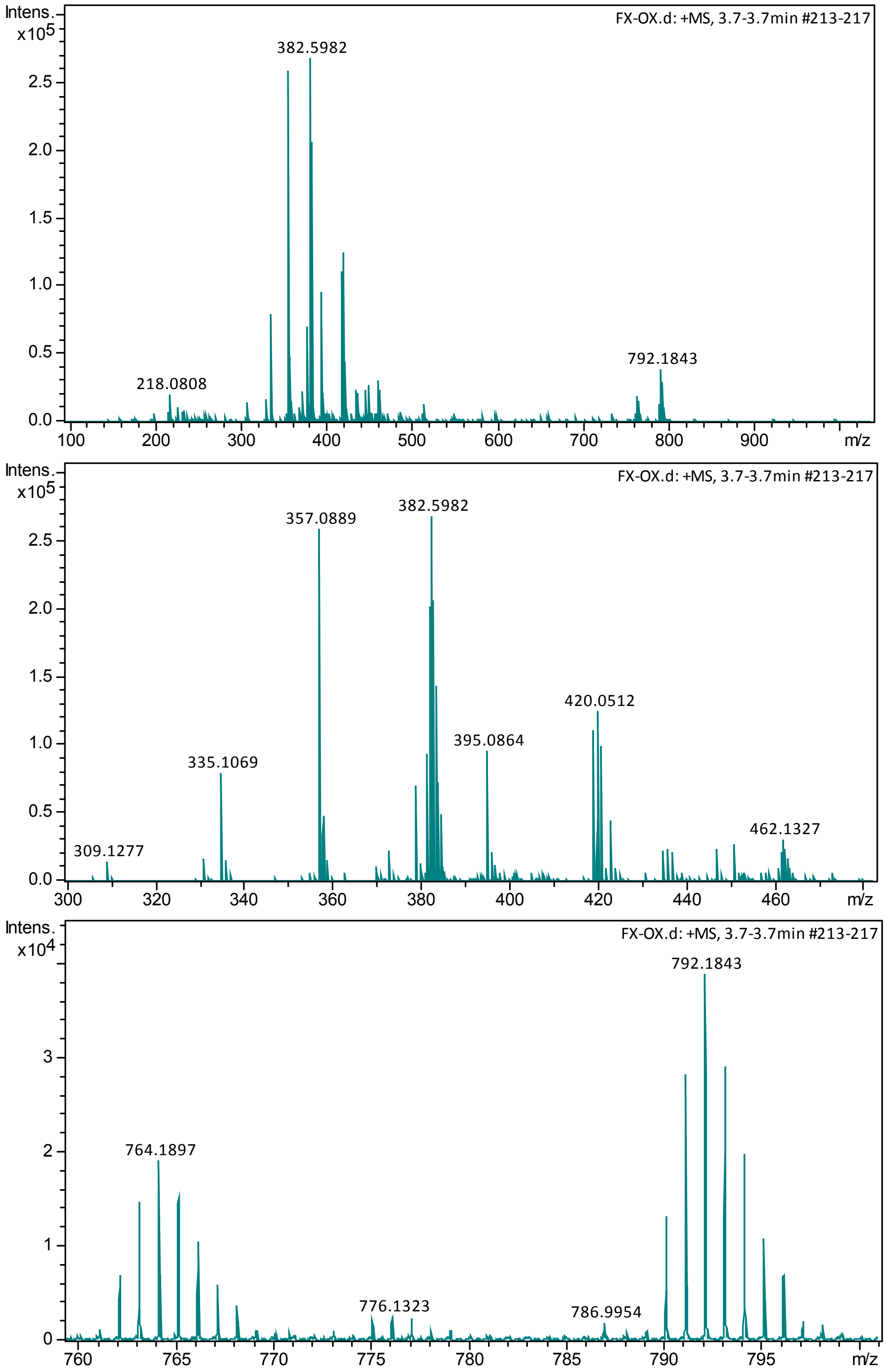

Figure S7. ESI-MS spectrum of sample **g** (1 mM oxaliplatin and 1 mM penicillin G) after incubation at 310 K for 24 h. Sample was diluted by 100-fold and then low resolution ESI-MS was recorded.
